# Within-Bolus Variability of the Penetration-Aspiration Scale Across Two Subsequent Swallows in Patients with Head and Neck Cancer

**DOI:** 10.1007/s00455-017-9814-2

**Published:** 2017-06-07

**Authors:** Johanna Hedström, Lisa Tuomi, Mats Andersson, Hans Dotevall, Hanna Osbeck, Caterina Finizia

**Affiliations:** 1 Department of Otorhinolaryngology Head and Neck Surgery, Institute of Clinical Sciences, Sahlgrenska Academy at the University of Gothenburg, Sahlgrenska University Hospital, 413 45 Gothenburg, Sweden; 2 Department of Radiology, Institute of Clinical Sciences, Sahlgrenska Academy at the University of Gothenburg, Sahlgrenska University Hospital, 413 45 Gothenburg, Sweden

**Keywords:** Deglutition disorders, Dysphagia, Deglutition, Head and neck neoplasms, Videofluoroscopy

## Abstract

To compare two consecutive swallowing attempts to study if there is a difference in Rosenbek’s penetration-aspiration scale (PAS) scores between the first and second swallowing attempt of the same bolus type in videofluoroscopic examination of swallowing (VFS). Additional aims include reflecting on which bolus sizes and consistencies are the most relevant to include in further studies for head and neck cancer (HNC) patients. The VFS for 38 patients curatively treated for HNC was studied. All included patients showed swallowing difficulties (PAS ≥ 2). The examination protocol included two swallows each of six different boluses: 3, 5, 10, 20 ml thin, 5 ml mildly thick, and 3 ml of extremely thick liquid. All boluses were compared between the first and second swallowing attempt with regard to PAS scores. No statistically significant differences in PAS were found between the first and second swallow for any of the boluses in this study on group level. For 20 ml thin and 3 ml extremely thick liquid, there were low Intra-Class Correlations, indicating a low within-bolus agreement. The greatest within-bolus differences were found for 20 ml thin, 5 ml mildly thick and 3 ml extremely thick liquid, which demonstrated high intra-individual coefficient of variation (0.458–0.759). The data of this study show a high within-bolus variability of the PAS score between two subsequent swallows for all different consistencies. In order to assess swallowing safety, the highest PAS score for each bolus type is suggested for use in studies of HNC patients.

## Introduction

Videofluoroscopic examination of swallowing (VFS) is a common method for assessment of swallowing function. There are different ways of interpreting the examinations, where rating scales are one option to describe the degree of dysfunction [1–3]. Studies often include biomechanical measures such as hyoid, larynx, and upper esophageal sphincter movement [4–6]. The interpretations always include some measure of penetration or aspiration [4–9], such as Rosenbek’s penetration-aspiration scale (PAS) [3]. Aspiration may lead to pneumonia, and maximum PAS has previously been shown to predict aspiration pneumonia [10]. Pneumonia occurs in up to one in four head and neck cancer (HNC) patients following concurrent chemoradiotherapy [11, 12]. In comparison, the incidence of pneumonia is about ten percent in a non-cancer population [12]. Accurate estimation of penetration or aspiration in HNC patients is therefore an important objective of VFS.

In VFS, different bolus sizes and consistencies are tested. Protocols and number of swallowing attempts differ between studies. Often, but not always, two or more swallowing attempts are performed for each bolus volume and consistency [4–6]. Table 1 lists VFS protocols used in a sample of studies of dysphagia in HNC patients. The practice of interpreting the results of VFS varies between studies. There is no consensus on whether to use the mean value from several swallowing attempts, to choose one particular swallow or analyze all swallows [4–6, 13–15]. For example, Frowen et al. indicate that data taken from the mean of several swallows may not be an accurate representation of swallowing function, due to within-bolus variability [4]. Reporting the mean of several swallows is only valid when there are no or small differences between the swallowing attempts [4]. This is not always the case, especially in cohorts of patients with dysphagia. However, few studies exist which address this matter. Therefore, in terms of penetration-aspiration events, it is of great interest to study the relevance of the boluses used, in order to determine which boluses are most clinically relevant, and which are of most interest to use in further studies of HNC patients.

**Table 1:** Boluses used in a selection of studies using videofluoroscopic examination of swallowing

	Logemann [5]	Frowen [4]	Frowen [13]	Rudberg [6]	Lee [7]	Starmer [18]	Mortensen [8]	Schwartz [27]	Kraaijenga [25]	Logemann [14]	Pauloski [15]
Swallows per bolus	2	3	2	2	1	1	1	1	1	2	2
1 ml thin	−	−	−	−	+	−	−	−	−	+	+
3 ml thin	+	+	+	+	+	−	+	−	+	+	+
5 ml thin	+	−	−	−	+	+	+	−	+	+	+
10 ml thin	−	−	−	−	+	+	+	+	−	+	+
20 ml thin or cup sips thin	−	−	+	+	−	+	−	−	−	+	−
3 ml thick	−	−	+	+	−	−	−	−	−	−	−
5 ml thick	−	−	+	+	−	−	−	−	−	−	−
10 ml thick	−	−	−	−	−	−	−	−	−	−	−
Pudding/paste/semi-solid	3 ml	3 ml	−	−	−	Tea-spoon	−	Tea-spoon	3 ml	3 ml	3 ml
Cookie/other solid food	−	−	−	−	−	+	+*	+	+	+	+
Total no. of swallows	6	6	8	8	4	5	4	3	4	14	12
Which bolus is analyzed	All	All, recommends 2nd	2nd	All	N/A	N/A	N/A	N/A	N/A	All	All

+ = yes

− = no

* Other solid food: carrot gratin

The aim of this descriptive study was to investigate the variance in PAS score between two consecutive boluses of the same volume and consistency in HNC patients with dysphagia. The choice of boluses for assessment of swallowing function, in patients treated with curative radiotherapy/chemoradiotherapy for HNC, in a clinical and research setting will be discussed.

## Materials and Methods

### Subjects

Patients with diagnosed HNC presented at the weekly multidisciplinary tumor board meeting at Sahlgrenska University Hospital Gothenburg Sweden were identified and judged as eligible for inclusion in the study if they reported swallowing problems at least 6 months after oncologic treatment. The inclusion criteria were as follows: patients diagnosed with cancers of the tonsil, base of tongue, hypopharynx or larynx, treated with curative external beam radiation therapy (EBRT) ± brachytherapy or chemotherapy, and having undergone VFS 6–36 months post oncologic treatment for HNC. The absolute inclusion criterion for the study was PAS ≥ 2 on any swallow, since we aimed to only evaluate patients with abnormal swallowing function according to PAS. Patients with a normal swallowing function (PAS = 1) were therefore excluded (*n* = 47). Patients having undergone surgical treatment, tracheotomy, or previous oncological treatment prior to HNC diagnosis, as well as patients with neurological or neuromuscular disease were also excluded from the study.

The study subjects were examined regarding dysphagia. The follow-up included VFS to evaluate swallowing function, where swallow function was analyzed and the degree of swallowing problems was quantified using the PAS.

### Videofluoroscopic Examination of Swallowing

Videofluoroscopic examination of swallowing (VFS) was performed using a multipurpose fluoroscopy system (Siemens Artis, Erlangen, Germany), with digital storage of high-resolution images (video matrix 1024 × 1024) at a rate of 15 frames per second. The patients were examined seated in the lateral position and the field of view included the tip of the tongue anteriorly, the pharyngeal wall posteriorly, the soft palate superiorly, and the seventh cervical vertebra inferiorly. Gastrointestinal radiologists trained in functional assessment of swallowing performed the examinations in cooperation with a speech-language pathologist (SLP). Six boluses were observed; 3, 5, 10, and 20 ml of thin, 5 ml of a mildly thick, and 3 ml extremely thick liquid. All bolus volumes were measured by syringe and placed into the patient’s mouth via syringe or spoon. For all boluses, except for 20 ml thin liquid, the patients were instructed to hold the bolus in their mouth until directed to swallow. For the 20 ml thin liquid, the patients were instructed to drink freely from a cup at a pace of their own choice. The patients were instructed to sip water to clear their pharynx between swallows. Detailed bolus description is presented in Table 2, where each consistency is also described with the standardized terminology according to The International Dysphagia Diet Standardisation Initiative (IDDSI) [16]. Swallowing of each bolus was performed twice. Not all patients were able to complete two swallowing attempts of each bolus. A patient may have refused to attempt one or both trials of a bolus; the speech-language pathologist also may have judged it as too great a clinical risk of excessive aspiration to make a second swallowing attempt of the bolus during the videofluoroscopic examination. Thus, for the safety of the patient, if the patient demonstrated a high degree of aspiration (e.g., PAS 7–8) on the first attempt of the bolus, no second attempt was made. Boluses where only one swallowing attempt was made were excluded from the analysis.

**Table 2:** Detailed description of the boluses used in the present study

	Bolus size and consistency (level according to the IDDSI framework [16])	Contrast
Bolus 1	3 ml thin liquid (0)	Mixobar colon 1 g Ba/ml mixed with equal amount of water
Bolus 2	5 ml thin liquid (0)	
Bolus 3	10 ml thin liquid (0)	
Bolus 4	20 ml thin liquid, drink freely (0)	
Bolus 5	5 ml mildly thick (2)	Omnipaque 300 mg I/ml. 20 ml Omnipaque mixed with 2 ml instant thickener
Bolus 6	3 ml extremely thick (4)	Omnipaque 300 mg I/ml. 20 ml Omnipaque mixed with 15 ml instant chocolate pudding mix

### Analysis of VFS

A gastrointestinal radiologist performed blinded analysis of the VFS according to Rosenbek’s PAS (Table 3) [3]. The digital technique used in the VFS examinations allowed for detailed evaluation of the act of swallowing by the combined use of slow motion analysis frame-by-frame and of static images. PAS is an equal-appearing interval scale used to describe penetration and aspiration events, ranging from 1 (no material enters the airway) to 8 (material enters the airway, passes below the vocal folds and no effort is made to eject). The PAS is commonly used to evaluate the swallowing outcomes following treatment for head and neck cancer [7, 9, 17, 18]. This tool has been found to differentiate between normal and abnormal airway protection during swallowing in healthy and dysphagia patients, providing information of substantial clinical relevance [19]. The PAS was therefore chosen as the single outcome measure in this study.

**Table 3:** Rosenbek’s penetration–aspiration scale [3]

PAS score	Definition
1	Material does not enter the airway
2	Material enters the airway, remains above the vocal folds, and is ejected from the airway
3	Material enters the airway, remains above the vocal folds, and is not ejected from the airway
4	Material enters the airway, contacts the vocal folds, and is ejected from the airway
5	Material enters the airway, contacts the vocal folds, and is not ejected from the airway
6	Material enters the airway, passes below the vocal folds and is ejected into the larynx or out of the airway
7	Material enters the airway, passes below the vocal folds, and is not ejected from the trachea despite effort
8	Material enters the airway, passes below the vocal folds, and no effort is made to eject

### Statistical Analysis

Descriptive statistics were used to summarize the demographical and clinical characteristics of the study subjects. The distribution of the variables was given as mean, standard deviation (SD), median, minimum and maximum for continuous variables, and as numbers and percentages for categorical variables. PAS scores for the first and second swallow for each bolus were compared. Since the data were paired and the variables were in interval scale, Wilcoxon signed-rank test (WSR) was used as the statistical method. For comparisons of agreement between the first and second swallow, intra-class correlations (ICC) were calculated. For calculations of variability between the first and second swallow, intra-individual coefficient of variation (CV) was calculated. CV was used in order to evaluate within-patient consistency, the variability of the measures in relation to the population mean, where numbers between 0 and 1 were obtained. The CV should generally be low, i.e., close to 0, in order to demonstrate good within-patient consistency.

### Ethical Considerations

The study was conducted in accordance with the Declaration of Helsinki and was approved by the Regional Ethical Review Board in Gothenburg, Sweden. All participants gave their written informed consent before inclusion in the study.

## Results

Thirty-eight patients were included in the study. Patient characteristics and treatment information of the participants are listed in Table 4.

**Table 4:** Patient characteristics and treatment information

	Mean (SD) min–max
*Age*	63.7 (8.0) 44–80

	*n* (%)
*Gender*	
Male	26 (68)
Female	12 (32)
*Smoking habits*	
Current smoker	12 (31.6)
Stopped smoking >12 months ago	12 (31.6)
Non-smoker	14 (36.8)
*Tumor location*	
Tonsil	14 (37)
Base of tongue	13 (34)
Hypopharynx	4 (11)
Larynx	7 (18)
*Tumor staging*	
I	5 (13)
II	3 (8)
III	6 (16)
IV	24 (63)
*Oncological treatment*	
EBRT	8 (21)
EBRT + induction chemotherapy	5 (13)
EBRT + concomitant chemotherapy	20 (53)
EBRT + induction and concomitant chemotherapy	5 (13)
*Tumor free after primary treatment*	38 (100)

*EBRT* external beam radiation therapy

No statistically significant changes were found when comparing the first and second swallow for any bolus on a group level (Table 5). However, the ICC for 20 ml thin and 3 ml extremely thick liquid were low (0.27 and 0.09 respectively), indicating a low within-bolus agreement. For these boluses, high intra-individual CVs were found (45.8–75.9%).

**Table 5:** Results from the calculation of the within-bolus changes

	PAS swallow 1	PAS swallow 2	Change diff 2–1	Change diff 2–1	ICC (2,1)	Intra-individual CV
	Mean (SD)	Mean (SD)	Mean (SD)	*p* value (WSR)		
3 ml thin liquid	2.35 (1.78) n = 37	2.22 (1.55) n = 37	0.135 (0.948) n = 37	0.574	0.840	0.292
5 ml thin liquid	2.48 (1.59) n = 31	2.42 (1.29) n = 31	0.065 (0.892) n = 31	0.847	0.814	0.254
10 ml thin liquid	2.35 (1.10) n = 34	2.44 (1.08) n = 34	−0.088 (0.570) n = 34	0.563	0.863	0.168
20 ml thin liquid (drink freely)	3.76 (2.10) n = 34	3.68 (1.92) n = 34	0.088 (2.442) n = 34	0.906	0.270	0.458
5 ml mildly thick liquid	2.28 (1.56) n = 36	2.50 (1.92) n = 36	−0.222 (1.551) n = 36	0.501	0.609	0.458
3 ml extremely thick liquid	1.91 (1.68) n = 34	1.68 (1.15) n = 34	0.235 (1.939) n = 34	0.691	0.090	0.759

*ICC* intra-class correlation coefficient, *CV* coefficient of variation, *WSR* Wilcoxon signed-rank test

Due to aspiration or inability to swallow in the first attempt, the patient did not perform the second swallow, with the consequence that no bolus size or consistency was completed twice for all patients

**Fig. 1:**
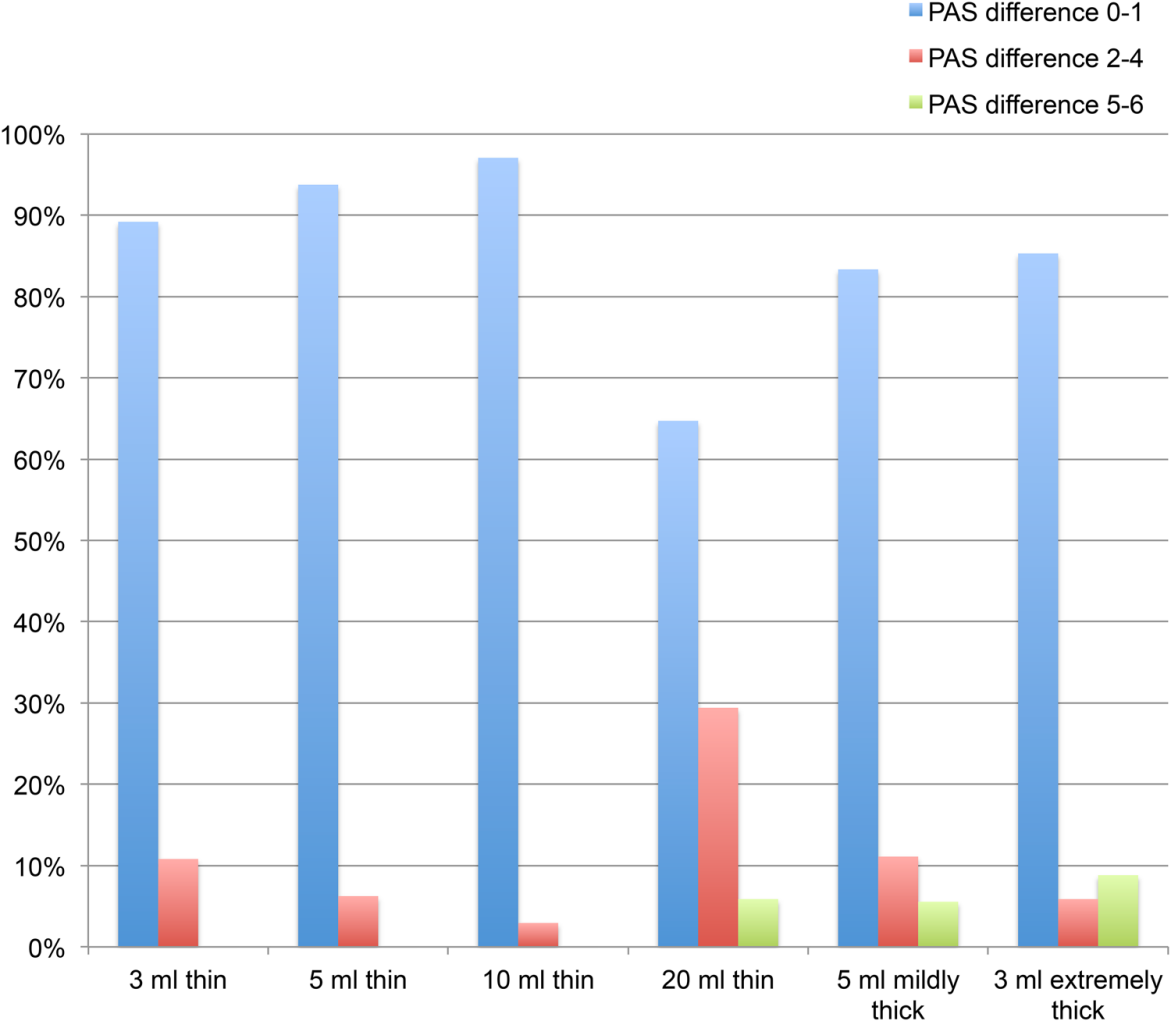
Differences in PAS values for all boluses between the first and second swallow. Proportion of differences divided into PAS difference 0–1, 2–4, and 5–6

Figure 1 demonstrates the magnitude of the difference between the first and second swallow for each bolus. Twenty ml thin, 5 ml mildly thick and 3 ml extremely thick liquid demonstrated the largest between-swallow differences. A 5–6 point difference in PAS was seen in 5.9, 5.6, and 8.8% of the subjects, respectively, when comparing the first and second swallow for these boluses. The rate of patients with a difference in PAS scores greater than two scale levels for the above mentioned boluses were 35.3, 16.7, and 14.7%, respectively.

## Discussion

The results of this study showed no statistically significant differences in PAS between the first and second swallow for any of the boluses on a group level. However, our data show differences in PAS score between two subsequent boluses on an individual level and between different volumes and consistencies. For the 20 ml thin liquid, 5 ml mildly thick liquid, and 3 ml extremely thick liquid, low ICC and high CV indicated that significant variability exist between the swallowing attempts of these boluses.

In studies using VFS, often several boluses are tested and the bolus consistencies and sizes differ through different studies, making interpretation and comparisons difficult to perform. The study by Frowen et al. is, to our knowledge, the only study that reports on measures of swallowing in terms of validity, reliability, and stability in HNC patients [4]. Stability refers to the level of agreement of two or more swallowing attempts performed in the VFS, i.e., if the score remains unchanged through multiple swallows, or if there are differences between the swallowing attempts of the same bolus. Reliability refers to whether a tool is dependable and measures the trait of interest, taking into account both agreement and consistency. Also, the swallowing measure needs to be valid, meaning that it measures what it is intended to measure. The study by Frowen et al. compared variables in swallowing for three consecutive swallowing attempts [4]. For both liquids and semi-solids, there were statistically significant differences across the three attempts, where either the first or the third swallow was the attempt that differed compared to the other two. Their recommendation is to use the second swallow, which provides a measure that more accurately represents the true score than the mean value [4]. In addition, the radiation exposure time must be taken into consideration when deciding on the number and types of boluses in the VFS protocol, as discussed by Martin-Harris et al. [20].

The high variability between swallows of 20 ml thin liquid is likely explained by the greater stress to the swallowing mechanism that this bolus poses compared to the smaller boluses. When drinking freely, even slight and/or intermittent incoordination between subsequent swallows may result in aspiration. High variability was also noted for the 5 ml mildly thick and 3 ml extremely thick boluses. This could be due to the fact that the patients often needed to swallow several times for the thicker liquids, which may result in aspiration in the same manner as for drinking freely. The large variability demonstrated in several of the boluses indicates a possible difference, which was not detected in the statistical analysis. When assessing the safety of the patient’s swallowing function, any tendency to aspirate is important to detect and therefore the “worst” PAS may be the most relevant variable to report.

Previous studies demonstrate that thicker consistencies generally result in less penetration/aspiration (lower PAS scores), both in normal subjects and patients following HNC treatment [21–23]. This demonstrates that it is important that different consistencies are represented in studies using VFS, in order to properly assess the impairment and allowing for the possibility to advise patients on which consistencies are safe to swallow. When selecting the appropriate boluses in VFS, the purpose of the study must be considered. In addition to PAS, other variables (e.g., kinematic, durational and structural variables) are important for identification of disturbances in swallowing physiology and for patient management. In the present study, we chose to use the bolus set of six types based on our previous experiences and standard procedures when examining patients with head and neck cancer.

The present data indicate that the thin liquid boluses of 3, 5, and 10 ml demonstrate quite similar PAS values, ranging from 2.22 to 2.48. ICC for these boluses ranges from 0.814 to 0.863 and intra-individual CV ranges from 0.168 to 0.292. Since these boluses demonstrate similar results, it may not be required to use all three during VFS. Martin-Harris et al. reported in their study that a 5 ml thin liquid together with a 5 ml mildly thick liquid contribute to the detection of swallowing impairment and should always be included in a VFS protocol [20]. Five ml thin liquid has previously been reported as a bolus that patients are able to swallow in all stages of radiotherapy treatment for HNC [24]. This bolus size is also common to include in studies using VFS [5, 7–9, 14, 18]. To allow for the comparisons between studies, the 5 ml thin liquid is a bolus that should be used in future studies. Additionally, 20 ml thin liquid is a bolus size, which proximately represents a normal situation, since the subjects are instructed to drink this bolus freely from a cup. Rogus-Pulia et al. chose bolus sizes and consistencies in order to represent a wide variety [23]. For this reason, the 5 ml mildly thick liquid and the 3 ml extremely thick could be used, similar to protocols used in other studies [4–6, 13–15, 18, 25–27].

This study is limited by the sample size. Possibly, a larger sample might have yielded statistically significant differences between some of the consecutive swallows. However, the sample size in this study is in relation to previous studies in the field [4, 5, 14, 23]. Another potential limitation is that the boluses where only one swallowing attempt was made were excluded from the analysis. Additionally, if the patient demonstrated a high degree of aspiration (PAS 7–8) on the first swallowing attempt of the bolus, no second attempt was made for the safety of the patient. Therefore, these patients were not included in the analysis. This may have introduced bias towards less variance in PAS within-bolus types. Our aim was to study the variance of PAS within-bolus types in symptomatic head and neck cancer patients with signs of disturbed swallowing function so the conclusions cannot be generalized to other patient groups.

In conclusion, the results of this study show that there is a variation in PAS between two consecutive swallows of similar boluses in a quite large portion of individuals treated for head and neck cancer. Future recommendations for assessment of swallowing with VFS in HNC are therefore to present the same bolus at least twice. Due to the high variability, reporting the highest PAS value is needed to assess the swallowing safety in all boluses. With the aim of testing the safety of swallowing: The boluses suggested for use in VFS examinations are 5 ml thin, 20 ml thin, 5 ml mildly thick, and 3 ml extremely thick liquid consistency. Further methodological studies are needed in order to determine which types of boluses are most useful in VFS studies in HNC patients.
